# Facilitators and barriers of implementing and delivering social prescribing services: a systematic review

**DOI:** 10.1186/s12913-018-2893-4

**Published:** 2018-02-07

**Authors:** Julia Vera Pescheny, Yannis Pappas, Gurch Randhawa

**Affiliations:** Institute for Health Research, University of Bedfordshire, Luton, UK

**Keywords:** Social prescription, Implementation, Delivery, Community care, Community referrals

## Abstract

**Background:**

Social Prescribing is a service in primary care that involves the referral of patients with non-clinical needs to local services and activities provided by the third sector (community, voluntary, and social enterprise sector). Social Prescribing aims to promote partnership working between the health and the social sector to address the wider determinants of health. To date, there is a weak evidence base for Social Prescribing services. The objective of the review was to identify factors that facilitate and hinder the implementation and delivery of SP services based in general practice involving a navigator.

**Methods:**

We searched eleven databases, the grey literature, and the reference lists of relevant studies to identify the barriers and facilitators to the implementation and delivery of Social Prescribing services in June and July 2016. Searches were limited to literature written in English. No date restrictions were applied. Findings were synthesised narratively, employing thematic analysis. The Mixed Methods Appraisal Tool Version 2011 was used to evaluate the methodological quality of included studies.

**Results:**

Eight studies were included in the review. The synthesis identified a range of factors that facilitate and hinder the implementation and delivery of SP services. Facilitators and barriers were related to: the implementation approach, legal agreements, leadership, management and organisation, staff turnover, staff engagement, relationships and communication between partners and stakeholders, characteristics of general practices, and the local infrastructure. The quality of most included studies was poor and the review identified a lack of published literature on factors that facilitate and hinder the implementation and delivery of Social Prescribing services.

**Conclusion:**

The review identified a range of factors that facilitate and hinder the implementation and delivery of Social Prescribing services. Findings of this review provide an insight for commissioners, managers, and providers to guide the implementation and delivery of future Social Prescribing services. More high quality research and transparent reporting of findings is needed in this field.

**Electronic supplementary material:**

The online version of this article (10.1186/s12913-018-2893-4) contains supplementary material, which is available to authorized users.

## Background

Psychosocial problems impact on the health and wellbeing of people [1]. Primary care staff may feel overwhelmed and not equipped to handle the psychosocial problems that primary care patients present with [2, 3]. The commonly available options for patients presenting psychosocial problems are medication, psychotherapy (cognitive behavioural therapy), and counselling [4]. Despite the potential benefits and policy attention, third sector (community, voluntary, and social enterprise sector) support to address the wider determinants of health in primary care often remains underused due to weak or non-existent links between the two sectors [5].

Social Prescribing (SP) is a relatively new approach in primary care that promotes partnership working between the health and the third sector [5]. Furthermore, SP expands the range of options available to General Practitioners (GPs), as it creates a formal means of enabling GPs to link patients with non-medical sources of support within the third sector [2, 6]. There is no definition of what sources of support constitute SP, examples of services and activities include art therapy, walking and reading groups, exercise classes, nature-based activities, and volunteering, as well as support with employment, debt, housing, and legal advice [3, 7]. The current health policy and guidelines, such as the English NHS’s 5 Year Forward View [8], the Social Value Act [9], and the National Institute for Health and Care Excellence (NICE) guidelines [10], are supportive of elements and approaches inherent to SP models.

SP models with different referral routes exist, for example, primary and social care patients may self-refer or be referred by a healthcare or other professional. Models may include a navigator (also termed a facilitator, referral agent/worker, coordinator, and social prescriber). The navigator’s role is to identify the non-medical needs of patients referred to a SP service and to refer, or signpost, them to sources of support within the third sector. Workshops hosted by Bromley Primary Care Trust (PCT) established the following six SP models [6]:

- Model 1: Information service
  This service is an information only service, with advertising and directory access to SP in a primary care practice.
- Model 2: Information service and telephone line
  This service advertises SP on leaflets and notice boards in a primary care practice. Based on this information, patients can self-initiate a telephone discussion with a worker.
- Model 3: Primary care referral
  Primary health care professionals assess patients during consultation and refer them to SP services if appropriate, for example if patient have non-clinical issues and require psychosocial support. Referrals to SP services are opportunistic.
- Model 4: Practice based generic referral worker:
  Primary care patients can be referred by health workers, or self-refer to a SP link worker. Clinics are held in the GP surgery, so that it can act as a “one stop shop”.
- Model 5: Practice based specialist referral worker:
  A specialist worker works from primary care practice and patients can be referred through primary care referral or self-referral. Direct advice and specific services, such as Citizens Advice, may be offered, as well as referral or signposting onwards.
- Model 6: Non-primary care based referral worker:
  Patients are referred to an external referral centre by primary care practice staff, offering one-to-one facilitation, for example an outreach service or set in the community.

In addition to these six models, Kimberlee et al. (2014) delineates SP interventions into the following four types: signposting, light, medium, and holistic. More information on these four types can be found somewhere else [11]. It is clear from the literature, that as of yet, there is no agreed definition of SP and different models exist. It is likely, that different models face different challenges during the implementation process and delivery of the service, due to the involvement of different pathways, organisations, and stakeholders. For instance, referred patients may be more likely to take up an activity when a supportive structure, i.e. a navigator, exists [2, 3].

Previous reviews have assessed the effectiveness of SP [7, 12, 13]. These reviews consistently found little good quality evidence [7, 12–14]. Randomised controlled trials are considered as the most reliable method, the ‘gold standard’, of determining effectiveness of interventions [15]. Although high quality research on the effectiveness of non-pharmacological and complex interventions, such as SP, is essential to inform policy and practice, outcome evaluations in isolation leave many important questions unanswered [16]. Effect sizes do not provide policy-makers with information on factors predicting or hindering implementation success, the processes of implementation, and how contextual factors influence the delivery and outcomes of interventions [16]. The value of process evaluations of complex interventions, as a complement not substitute to outcome evaluations, has been recognised and process evaluation has been added to the updated guidance of the Medical Research Council (MRC) in 2008 [17]. The MRC defines process evaluation as a study that examines the implementation, mechanisms of impact, and contextual factors to understand the functioning of an intervention [16]. Implementation research can consider any aspect of implementation, including factors that hinder and facilitate the implementation and delivery of an intervention [18]. Previous research found that a number of common facilitators and barriers emerged across integrated care pilots in the UK [19, 20]. Factors that appeared to be particularly relevant for integrated care include existence of training for new staff, staff stability, physician involvement, and information technology systems [19]. In addition, many of the barriers and facilitators to the implementation of integrated care pilots were found to be those of any large-scale organisational change [19, 20]. Examples of such factors include quality of leadership at the top and within groups, flexibility of organisational culture, and the availability of resources.

The identification of barriers and facilitators to SP programmes in the UK can inform policy and practice, and potentially improve future implementation of such programmes [19, 21]. A well-led implementation process is important as it influences the delivery and outcomes of a programme [16]. If a health intervention is not implemented sufficiently due to encountered barriers, the delivery process can be disrupted and negative outcomes can occur [16, 21]. To the best of the authors’ knowledge, there is no systematic review on factors that hinder and facilitate the implementation and delivery of SP programmes. It is imperative for service commissioners and providers to understand the operational facilitators and barriers in relation to specific SP models to inform future service provision. Furthermore, the synthesis of the available evidence on factors that hinder and facilitate the implementation of specific SP models, promotes an understanding of how the findings can be compared to those of other integrated care pilots and large-scale organisational change.

The current review focused on facilitators and barriers to the implementation of SP models based in general practice involving a navigator. In this model, general practice staff refers patients to a navigator, who assess the non-medical needs of patients and refer, or signpost, them to sources of support within the third sector.

### Study objective

The objective of this review was to identify factors that hinder and facilitate the implementation and delivery of SP services based in general practice and including a navigator in the UK.

## Methods

### Protocol

Methods of the analysis and inclusion criteria were specified in advance and documented in a protocol. The protocol is part of a PhD study and will be made available once the thesis is made publicly accessible.

### Study design

We conducted a systematic literature review of studies assessing SP services based in general practice and involving a navigator. Data synthesis built on a narrative synthesis, using thematic analysis for categorising data. Narrative synthesis is a commonly used method to synthesise data in the context of a systematic review [22, 23]. As thematic analysis provides the means of identifying relevant themes (based on the review question) across large and diverse bodies of research [24], this approach was employed to synthesise the findings. As this research did not involve human subjects or animals, we did not seek an ethics opinion.

### Inclusion and exclusion criteria

SP is an emerging field of research and there is limited evidence on SP interventions in the UK to date. To maximise the inclusion of the available evidence on factors that hinder and facilitate the implementation and delivery of SP interventions, this review was not limited to a specific study design. Hence, all types of study designs, qualitative, quantitative, and mixed-methods, were included in the review. As the review focused on SP services that are based in general practice and involve a navigator (see description in introduction), studies that did not meet both criteria were excluded from the review. Studies involving SP services had to be implemented in the United Kingdom (UK), and so all SP services outside of the UK were excluded. Any studies that met the inclusion criteria and referenced any factor that hindered or facilitated the implementation or delivery of SP services were included in the review.

### Search methods

Several articles and evaluation reports of SP services were identified through an initial exploratory online search using the search engine ‘Google’ and the electronic database **‘**Web of Science’. To get familiar with relevant terms, the authors attended steering group meetings supporting the implementation of a SP service in England, attended workshops on SP, and reviewed the search strategies of previous literature reviews related to SP. This, together with the objective of the review, informed the terms of the search strategy and supported the development of an inclusive and rigorous search strategy. Searches were conducted in June and July 2016. Detail of the search strategy is provided in Additional file 1.

Eleven databases were searched from their start dates to July 2016: CINAHL (The Cumulative Index to Nursing and Allied Health Literature), ASSIA (Applied Social Sciences Index& Abstracts), British Nursing Index, Web of Science, Cochrane library, Medline, PsychInfo, Sport Discuss, HMIC (Health Management Information Consortium), and University of York Centre for Reviews and Dissemination (DARE, NHS EED, HTA). The searches were limited to literature written in English. No date restrictions were applied.

To identify relevant evaluations in UK settings, the websites of the following organisations were searched:

- The Kings Fund
- The Health foundation
- NESTA
- NICE
- Nuffield Trust
- Department of Health

Additionally, grey literature was searched in OpenGrey, Google, and Google Scholar. The search terms “social prescribing” and “social prescription” were used to identify the grey literature. The grey literature search was conducted in June and July 2016. In addition, the reference lists of all relevant studies, reviews, and reports were searched.

### Selection of studies

After eliminating the duplicates (studies that were identified more than once by the search engines), an initial screening of titles, abstracts, and summaries (if applicable) was undertaken by one reviewer with a random 25% of the sample checked by a second reviewer. The full text was obtained for all the records potentially meeting the inclusion criteria (based on the title and abstract/summary only). In a second step, one reviewer screened the full papers against the inclusion criteria, with a random 25% of sample checked by a second reviewer. Any discrepancies were resolved through discussion between the first and second reviewers and, if consensus was not reached, with a third reviewer.

### Data extraction

Data extraction of the included studies was conducted by one reviewer and checked by a second reviewer, using data extraction forms tailored to the requirements of the review. The extraction form was tested on three included papers and, where necessary, it was revised to ensure it can be reliably interpreted and can capture all relevant data from different study designs. Extracted data included authors, year of study/report, type of paper (e.g. journal article, annual evaluation report), study design, description of the SP service (model descriptions, referrers, target group), study sample, and factors that facilitate and hinder the implementation and delivery of SP services. Any discrepancies were resolved through discussion between the first and second reviewers and, if consensus was not reached, with a third reviewer.

### Methodological quality assessment

From the literature review it is clear that the evidence base of SP services in the UK is weak. Therefore, to capture the available evidence on the implementation and delivery of SP services, for which more rigorous studies are lacking, no exclusion on the basis of methodological quality was made. The authors recognised that some studies may have poorly documented methodological quality but may have contextually rich detail that contributes to the overall narrative synthesis. Assessment was undertaken to ensure transparency in the process and to make the limitations of poor quality studies explicit to improve future research.

The methodological quality of included studies was appraised independently by two reviewers using the Mixed Methods Appraisal Tool Version 2011 (MMAT-V 2011) [25]. The conference paper by Polley et al., [28] was not quality appraised, as the MMAT-V 2011 was not designed for conference papers.

### Data synthesis

Findings from included studies were synthesised narratively. The ‘Guidance on the Conduct of Narrative Synthesis in Systematic Reviews’ was used to advise the narrative synthesis in this study [24]. First, a preliminary synthesis was conducted to develop an initial description of the findings of included records and to organise them so that patterns across records could be identified. This followed the iterative approach of a thematic analysis, where multiple ideas and conclusions across a body of literature were categorised into themes [26]. The created themes were reviewed and refined throughout the process.

## Results

**Fig. 1:**
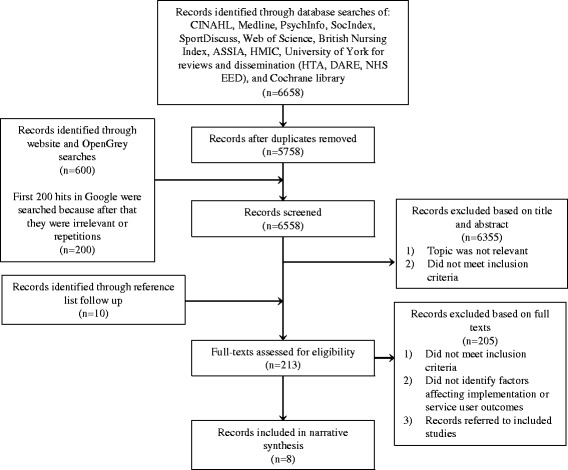
Adapted study selection flow diagram based on PRISMA [27]

In total, the titles and abstracts/summaries of 6558 records were screened. Of these, 213 records were considered potentially eligible and were assessed in full text. Eight records met the inclusion criteria of the review. An adapted PRISMA (Preferred Reporting Items for Systematic Reviews and Meta-analyses) flow-chart of study selection is presented in Fig. 1 [27].

In total, the included studies comprised of one conference report [28] and seven evaluation reports [29–35]. The publishing date of the Age UK report is unknown [34]. All other included records were published in the last 5 years [28–33, 35], highlighting that SP is a relatively new phenomenon in the UK. None of the included records were journal articles and factors that hinder and facilitate the implementation and delivery of Social Prescribing services were not the main focus of any included record. This supports previous claims that there is a dearth of research adequately exploring facilitators and barriers to the implementation and delivery of SP services in the UK. Summary details of the reviewed literature are available in Table 1.

**Table 1:** Overview of studies and reports included in the review

First author and year	Type	Study design	Qualitative data collection tools	Objectives	Participants in qualitative research	Intervention description
Dayson 2013 [26]	Evaluation report	Qualitative study	Interviews	To identify emerging lessons from the implementation and operation of the Rotherham SP programme	Service users, public sector stakeholders, project staff, voluntary and community organisations	General Practitioners identify and refer primary care patients with complex long-term conditions and carers of case-managed patients to a navigator who assesses the non-clinical support needs of patients and carers before referring them to appropriate services in the voluntary and community sector
Friedli 2012 [23]	Evaluation report	Mixed methods study	Interviews	To build local evidence of the benefits of SP for patients and health professionals and to identify operational issues and solutions in running a practice based scheme	Service users, general healthcare professionals, navigators	General practice staff identifies and refers suitable patients to a navigator who assesses the psychosocial needs of patients and refer them to appropriate sources of support in the third sector
The Health Foundation 2015 [24]	Evaluation report	Mixed methods study	Interviews	To fill the gaps in the existing evidence base	Service users, navigators, GPs, community organisations, staff from City and Hackney CCG	General practice staff identifies and refer patients to a navigator employed by Family Action (FA). The navigator assesses the individuals’ needs to refer them to appropriate non-clinical community services delivered by 85 statutory and voluntary groups
Age UK nd [25]	Evaluation report	Not specified	Not specified	Not specified	Not specified	General Practitioners refer patients to local Age UK teams who assess the psychosocial needs of service users and refer them to appropriate services provided by Age UK
ERS Research and Consultancy2013 [21]	Evaluation report	Mixed methods study	Interviews	To assess the impact and achievements of the Social Prescription programme in Newcastle and to document lessons learned to inform further practice	Service users, healthcare practitioners, members of the steering group	Healthcare professionals refer patients with psychosocial needs to one of the five collaborating Linkwork Organisations (Age UK, HealthWorks, Newcastle Carers, Search, and West End Befrienders). Link worker from the Linkwork organisation assess the non-clinical needs of referred patients and either refer them to activities provided by them or refer them to other activities in the third sector to meet their non-clinical needs.
Farenden 2015 [22]	Evaluation report	Mixed methods study	Semi-structured telephone interviews and in-depth telephone interviews	1. Assess the impact of the pilot, for patients, volunteers, and General Practitioners 2. Analyse costs-benefits and social value 3. Outline key lessons, challenges, and successes 4. Discuss opportunities and risks 5. Present a business case with options for a future model	Service user, General Practitioners, practice manager	General Practitioners identify and refer patients with psychosocial needs to a navigator who assesses the psychosocial needs of patients and refers them to activities and services in the third sector. Six appointments of 45 min are offered to a referred patient. Once a patient is referred to an activity, the navigator follows-up the process, and if needed, offers further support. Navigators are volunteers with a background in helping people to meet their social or support needs.
Brandling 2011 [20]	Evaluation report	Mixed methods study	Semi-structured interviews, notes of navigators’ reflective diaries	1. To measure the impact on individuals’ mental wellbeing and specific issues identified at assessment 2. To assess user satisfaction with the social prescribing service 3. To understand the nature of engagement with a ‘New Routes’ service from different stakeholder perspectives 4. To provide case studies of New Routes that illustrate the type of work being conducted 5. To use research findings as they emerge to inform the conduct of the Social Prescribing service during the service development and research period 6. To use research findings as they emerge to inform the conduct and sustainability of the New Routes service	Patients, navigators, GPs	Healthcare professionals refer patients with social disengagement or low mood leading to a loss of connection to other people and the community, to the Social Prescribing programme (New Routes). Navigators assess patients’ non-medical needs in a one-hour appointment and connect them with appropriate sources of support in the third sector.
Polley 2016 [19]	Conference paper	Qualitative survey, discussions in mixed stakeholder groups	NA	The report provides a summary of the emerging themes of the pre-conference survey and discussions from the day	Relevant stakeholders	NA

### Outcomes

Findings from included records were classified in two major groups for this review: (i) facilitators, and (ii) barriers to the implementation and delivery of SP.

The qualitative research findings of seven mixed methods studies and one conference paper have been used for the thematic synthesis. The following section will present the facilitators and barrier by the identified themes.

### Facilitating factors

#### Implementation approach

Applying a phased roll out approach to implement SP interventions, i.e. changes are made over a period of time with a scheduled plan of steps, was identified as a facilitator to the implementation and delivery of SP [31]. It has the potential to support the development of new and effective partnerships between GP surgeries, navigators, and the third sector and allows time to develop a shared understanding about the programme and expectations between involved partners [29, 31]. It is important to plan a realistic ‘lead in’ time for setting up SP services, considering that it can take several weeks to set up initial meetings with GP practices [34].

#### Organisation and management

Organising a series of workshops to design and discuss a SP service prior to its implementation and standardised training for involved partners, briefings, and networking events to share best practice were identified as facilitators to implementation and delivery of SP services [30, 33]. Training for referrers on how to explain SP to patients, i.e. words and examples they can use, is likely to encourage referrals to SP services [32]. Regular steering group meetings, to discuss processes, arrange operational procedures, and react to challenges, facilitate the implementation and delivery of SP programmes [31].

In addition, flexibility, i.e. hearing what stakeholders need from the service and altering systems, processes, and communications accordingly, during the development, implementation, and delivery stage was identified as a facilitator to implementation and delivery [31, 35]. A flexible approach turned out to be effective particularly when working in partnership with GP surgeries, as each surgery is a unique organisation and may have different needs to implement and run SP [31]. A referral system for SP, for example, that fits with established referral systems and pathways in the general practice facilitates referrals to SP services [29, 34].

#### Shared understanding and attitudes

Shared understanding among clinical and non-clinical staff of what can be expected by each partner, the scope of the SP service, which patients to refer, how patients can be helped, and the capacity and skills offered by a navigator facilitates the implementation and delivery of SP services [31]. Shared understanding between partners from different sectors, commissioners, service users, and stakeholders, is crucial to manage expectations and to prevent tensions and disappointment during the implementation and delivery of SP services [28, 31]. Shared perspectives, attitudes, and understanding of the programme and strength of partnership are essential for effective partnership working between the health, community, and the third sector, which is a key principle of SP interventions [31]. To increase primary care patients’ understanding of SP interventions, the following approaches were recommended: a publicity campaign [32], working in collaboration with the practice manager to promote SP on TV screens in general practices [32, 34] and through the practice newsletter, the practice website, and information in waiting rooms [34].

#### Relationships and communication

Creating new relationships between partners based on reciprocity and trust may facilitate the implementation and delivery of SP services [31]. A good relationship between navigators and other partners (i.e. general practice staff and service providers), is particularly important, as it promotes effective communication [31, 34]. Feedback on service users’ journeys and outcomes to GPs and practice staff, via the navigator e.g. during regular meetings or a short periodic report, helps general practice staff to understand how patients progress after their referral [30, 31, 33, 34]. In addition, structured contact and regular communication between navigators and practice staff, served as a reminder for SP, encouraged a higher number of referrals, and ensured a greater appropriateness of referrals [30, 31].

Regular feedback and effective communication between the navigator and service providers in the third sector facilitates the implementation and delivery of SP services, as it allows to react to emerging challenges and promotes shared delivery and partnership working [33, 35].

#### Organisational readiness

Lessons learnt form the SP pilot in Brighton and Hove show that general practices need to be ‘Navigator ready’ before a navigator can start to work in a practice. The following is recommended by Farenden et al. [31] for a GP surgery to become ‘Navigator ready’:

1. It is important that the SP team meets the whole practice team (clinical and non-clinical staff) before SP commences. This could happen during a training session or practice meeting. The SP team should ensure they work flexibly when arranging a visit.
2. A partnership agreement needs to be signed between the SP service and the GP surgery hosting it.
3. GPs agree to make regular referrals to the SP service. Numbers depend on navigators’ capacity.
4. Navigators should be treated as a member of the primary care staff team. To ensure this happens, surgery staff need to:

- Understand the scope of the SP programme and the navigator’s role and skills
- Provide a room for the navigator, which are accessible for patients and allow meetings without interruptions
- Provide an induction including available staff facilities, safety procedures, computer login details, and telephone access
- Invite the navigator to relevant meetings
- Clarify how and when the navigator can contact the GP directly
- Provide a lead staff member who can answer queries relation to surgery systems and communications
- Provide a secure space for navigators to keep their files, working material, and confident records in the general practice

‘Navigator ready’ practices are crucial to facilitate the implementation of SP and to ensure that an effective and equitable service is delivered to service users [31].

A key lesson learnt from the SP programme in Maryfield is that GPs are more likely to make regular referrals to SP when the practice culture supports holistic and psychosocial approaches [32]. Moving away from the biomedical model of health towards a biopsychosocial model of health, considering alternatives to traditional medical interventions, and addressing wider determinants of health, i.e. considering social, psychological, and environmental determinants of health instead of focusing solely on medical needs, facilitate the implementation and delivery of SP services [32].

#### General practice staff engagement

Health professionals and practice staff engagement, involving regular referrals to SP, is a facilitator and crucial for the implementation and delivery of SP services [31]. Strategies that may encourage and maintain engagement of health professionals include feedback letters from navigators to prescribers, regular education events and training sessions, encouraging navigator attendance at surgery staff meetings, having information stalls within practice reception areas, and a brief and easy to complete referral form to reduce the workload for prescribers [31, 33, 34]. Furthermore, having SP champions based in general practice and Clinical Commissioning Groups (CCGs), fosters support, encourages regular referrals to the SP service, raises the profile, and perceived value of SP among general practice staff [28, 31, 34].

#### Support and supervision

The support of the practice manager is vital for arranging meetings with GPs, to build relationships between the SP team and the general practice, and to increase awareness about SP during the ‘lead in’ time, implementation, and delivery [34]. A supportive structure for navigators can facilitate the implementation and delivery of SP services, however a diverse nature of the support structure may require the adherence to multiple different interests which may have felt conflicting for navigators at some times [29]. A framework for the support that should be provided by navigators, facilitates the consistent delivery of SP services [30].

#### Infrastructure

A wide range of good quality third sector based services and activities, that are easily accessible with pubic transport, facilitate the implementation and delivery of SP services [31, 32].

### Barriers

#### Leadership and organisation

A collaborative multi-sector approach to project management, i.e. involving a diverse group of stakeholders, contributed towards a delayed implementation and delivery of SP [30]. The lack of a targeted approach to strategic and robust project management, to undertake all the coordination required for the programme, may result in less effective and delayed implementation and delivery of SP initiatives [30].

The absence of a robust risk management system, to be prepared for scenarios that could disrupt implementation and delivery, was identified as a further barrier to the implementation and delivery of SP [30].

Operating a SP service with volunteers as navigators may delay the implementation and requires a higher level of flexibility than is necessary with paid staff [31]. Changes to processes and procedures can take longer and may require more intensive support to be implemented than what would be expected of paid staff [31]. Another identified issue were inconsistencies in record keeping that resulted in extra work an costs for staff [31]. Furthermore, volunteer turnover is generally higher than paid staff turnover, with an average of one in three volunteers leaving the role within a year [31]. Frequent staff turnover disrupted the continuity of the delivery process and required resources to train new volunteers [31].

A lack of a partnership agreement between the SP service and the GP surgery hosting it, outlining the scope of the programme, the role, and what can be expected from each partner, was identified as a barrier [31]. It may lead to tensions, a mismatch of expectations, and different understandings of the SP programme between partners, which were identified as barriers to implementation [31]. The absence of a mutually agreed service level agreement, including the scope, model, data requirements, arrangements, and details of governance structure was identified as a further barrier [31]. It may lead to constant changes to the agreed model and data requirements, resulting in increased staffing costs for service management, navigator coordination, and data monitoring [31].

#### Implementation approach

A ‘go live dates’ approach to initiate SP in general practices, i.e. following set dates to initiate SP in surgeries, was identified as a barrier to the implementation and delivery of SP services [31]. Navigators and practice staff were rushed into hosting SP without building relationships and trust between partners, developing shared understanding of outcomes and expectations, agreeing mutually effective working practices, and ensuring the surgery is prepared to host a navigator [31]. Limited availability or lack of designated rooms for navigators in surgeries was identified as a key barrier to the implementation of SP models in which navigators are based in surgeries [31].

#### Economic climate and funding

In markets where there is high employee mobility, staff who are employed via temporary contracts to support SP pilots (e.g. navigators or project managers), may seek alternative more stable employment, as the future or prospect of their roles might be unclear [31, 35]. Limited resources may create a barrier to recruit highly skilled navigators, due to relatively low pay [28], and to engage service providers in the third sector, due to little available funding to support them [33].

#### Shared understanding

The lack of shared understanding of a SP service and pathway among stakeholders, including prescribers, navigators, service users, and service providers, was identified as a barrier to the implementation and delivery of SP services [29, 31, 33]. Lack of shared understanding may result in the lack of mutual trust between partners and prevent effective partnership working, a key element of SP [31]. Furthermore, limited understanding of the SP pathway among prescribers may result in uncertainty on how to explain SP to patients, which in turn may hinder referrals to SP services [29, 32]. It may also hinder the provision of consistent and fulsome information to patients, which may lead to wrong expectations towards SP [29]. Lastly, lack of referrers’ understanding may lead to large numbers of inappropriate referrals which hinder the delivery of the programme to the target group, and requires additional time (staff hours), which result in additional costs and delays [31].

#### General practice staff engagement

Low or no practice staff engagement is a key barrier to the implementation and delivery of SP, as the SP pathway starts with a referral from practice staff to the SP service [31]. Lack of trust in navigators, lack of time within busy consultations, lack of confidence to explore the social determinants of health, forgetting about the availability of SP, and scepticism about patients effectively attending activities in the third sector once referred, were identified as barriers to making referrals to SP programmes [28, 29, 31, 33].

#### Staff turnover

The continuity of the SP programme in City and Hackney was affected when two of the navigators left the SP project after the first year [33]. The CCG senior project lead officer left the SP project in Newcastle, which resulted in the loss of links to key personnel within the CCG and GP practices, delaying the delivery of the SP programme [30].

#### Patient engagement

No or low patient engagement is a major barrier to the implementation and delivery of SP services [29, 30, 32]. GPs felt that engaging patients is difficult because SP is a new way of working in general practice, which is difficult to explain, and patients do not understand the idea of SP when GPs explain it in a consultation [32]. Other reasons for disengagement in SP are: lack of interest in SP and scepticism around its potential benefit [29], patient entrenchment in medical solutions [29, 32], low motivation to move from contemplation to action [29], perceived threats to welfare benefits [32], fear of stigmatisation because of a link to mental health services and data collection tools such as the Warwick Edinburgh Mental Wellbeing Scale (WEMWBS) [29], lack of confidence [29], money issues, and transport issues to the prescribed services [29].

#### Infrastructure

There is a risk that available services and activities in the third sector may be cut below the level of service users’ needs, which could hinder the delivery of SP services. Navigators have reported difficulties to refer service users to appropriate services and activities because of reductions in scope and long waiting lists [31, 32].

The results of the systematic review are summarised in Table 2.

**Table 2:** Summary of identified facilitators and barriers to the implementation and delivery of SP services

Facilitators	Barriers
• A phased roll out implementation approach • Realistic planning of ‘lead in’ time to set up a SP service • Workshops to design and discuss SP services prior to implementation • Standardised trainings, briefings, and networking events for involved partners • Flexibility during the development, implementation, and delivery of a SP service • Shared understanding, attitudes, and perspectives of stakeholders • Good relationships and effective communication between stakeholders within and across sectors • SP champions in CCGs and general practices • Navigator ready general practices • A general practice culture that supports the biopsychosocial model of health • General practice staff engagement • A wide range of good quality third sector based service providers	• A ‘go live dates’ approach to implementation • Lack of partnership and service level agreements • A collaborative approach to project management, which results in the lack of a targeted approach to strategic and robust project management • Absence of a robust risk management systems • Volunteers as navigators • Staff turnover • Limited financial resources to fund service providers or secure a high salary for employed staff • Lack of shared understanding among stakeholders and partners • General practice staff disengagement • Patient disengagement • A reduction in available and suitable service providers in the third sector

#### Quality appraisal

Most of the included records failed to attain higher quality scores as a result of lack of detail on methodology. Most of the included evaluation reports lacked clear and well-focused objectives and did not provide detailed information on data collection tools, recruitment and sampling strategies, and data analysis methods. Methodological information tends to be spread over evaluation reports and can be found, for example, in footnotes in small print or in the Appendix. There is a lack of a structured and detailed methodology section in most evaluation reports of SP in the UK, which creates a challenge to the quality appraisal of available evidence. The quality scores of each study are presented in Table 3.

**Table 3:** Quality scores of included studies calculated using the MMAT-V 2011

First author and date	Overall quality score
Brandling 2011 [20]	***
Dayson 2013 Chapter 4 [26]	**
Farenden 2015 [22]	**
Friedli 2012 [23]	*
The Health Foundation 2015 [24]	*
ERS Research and Consultancy 2013 [21]	*
Age UK n.d [25]	–

Scoring metrics: ‘-’ = Further appraisal was not feasible as the answers to the two screening questions were ‘no’ or ‘can’t tell ‘*’ = 25%, ‘**’ = 50%, ‘***’ = 75%

## Discussion

### Main findings

To our knowledge, this is the first systematic review identifying factors that facilitate and hinder the implementation and delivery of SP programmes based in general practice. The review has identified a range of barriers and facilitators, which are summarised in Table 2. The following barriers and facilitators were found to be similar to those of other integrated care programmes: Relationships and communication between individuals and organisations [19], professional engagement [19], shared understanding [20, 36], support and training for staff in new roles [20], leadership [19, 37], staff stability [20], a phased roll out approach to implementation [20], and flexibility and permissiveness of organisational culture [19]. All these factors are similar to those identified by the Department of Health’s national evaluation of 16 integrated care pilots across England [19]. However, the following two themes were not identified by the Department of Health’s evaluation: Service level and partnership agreements and patient engagement. Previous research stresses the importance of patient-level factors (e.g. health-relevant beliefs, personality traits, motivation, and trust) for the implementation of health care interventions [38]. Patient-level factors impact on the outcomes of implementation efforts, as patients are active agents and consumers of healthcare [38]. Patient-level factors were identified as relevant factors for the implementation of integrated diabetes care in Ireland [39]. As found in this study, service level and partnership agreements seem to determine the level of shared understanding of stakeholders’ roles, values, and the intervention’s goals, scale, and scope. Hence, partnership and service level agreements may have a direct influence on shared understanding, which was identified as a relevant factor for implementation of integrated care pilots in the current and a previous study [19, 36]. In addition to the themes that are relevant to integrated care pilots in general, this study identified a theme that seems to be relevant to SP interventions explicitly: Local infrastructure. Given that SP interventions usually include service providers in the third sector to deliver care to service users, the local infrastructure was identified as a factor influencing the implementation process of SP interventions. Finally, two factors that are specific to SP models based in general practice and involving a navigator were identified: Navigator ready surgeries and the involvement of primary care practice managers in the development and implementation of the intervention.

Findings of this study indicate that many barriers and facilitators to the implementation of SP programmes are similar to those of other integrated care pilots and any large-scale organisational change [19]. However, some identified factors are specific to social prescription interventions, and specific to SP models. Hence, lessons learnt from the implementation process of other integrated care pilots can inform the implementation of social prescription interventions, but do not consider intervention specific factors such as the local infrastructure, navigator ready surgeries, and the involvement of practice managers. Developing an evidence base on facilitators and barriers to specific SP models allows policy makers, managers, and practitioners to promote facilitators and overcome specific potential barriers, to improve the implementation and delivery process of SP interventions.

Consistent with the findings of other reviews of SP services, this review has found that the quality of the majority of included studies was poor [7, 12–14]. The methodologies were often poorly reported, with sparse data on numbers of participants, a non-comprehensive sampling strategy, and a lack of information on the process of collecting and analysing data. In addition, the review found that all of the included studies and reports made reference to barriers and facilitators to the implementation and delivery of SP services, but none looked specifically at these factors. In addition, besides a comprehensive search strategy including a large number of databases, all included studies were identified through the grey literature search or screening of reference lists of relevant literature. Hence, there is a clear need for rigorously designed, analysed, and transparently reported research studies on barriers and facilitators to the implementation and delivery of SP services. Particular consideration should be given to the dissemination of research findings.

The publication date of the included AGE UK report is unknown [34]. All of the other seven included records were published in the last 5 years [28–33, 35], indicating that SP is a relatively new phenomenon in the UK.

### Relationships between identified facilitators and barriers

**Fig. 2:**
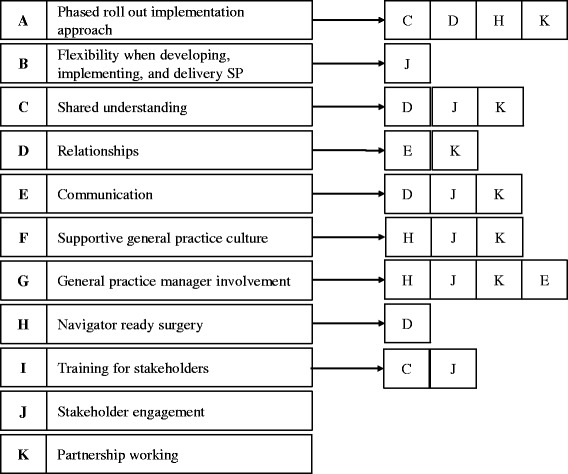
Interrelationship between facilitators to the implementation and delivery of Social Prescribing services

**Fig. 3:**
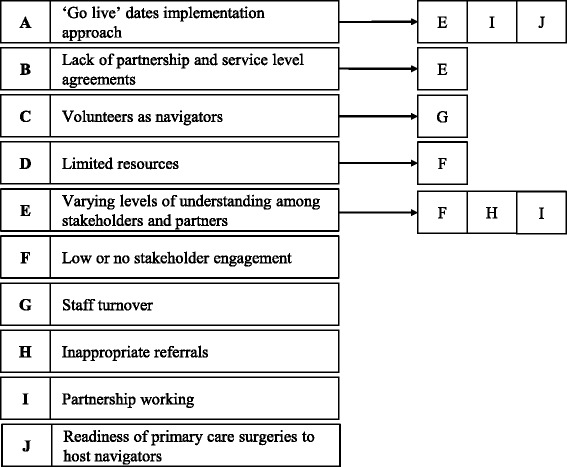
Interrelationship between barriers to the implementation and delivery of Social Prescribing Services

Through the thematic analysis, the interrelationship between identified facilitators and barriers was exemplified. For example, a phased implementation approach affects other factors that were identified as facilitators, namely shared understanding, relationships, and navigator ready surgeries. Figure 2 shows the identified interrelationships between facilitating factors and Fig. 3 shows the interrelationships between barriers. These interrelationships show that the implementation and delivery of SP services is a complex process. Barriers and facilitators can promote other barriers and facilitators or their relationship can be bidirectional, as for example between the following two facilitators: Relationships and communication (Fig. 2). Good relationships between partners seem to promote effective communication between them, and, in turn, effective communication between partners fosters good relationships.

### Limitations

There are important limitations to this review. A first limitation is the potential publication bias. Other studies may exist but have not been accepted or submitted for publication and therefore were not identified through the authors’ searches. Second, not all findings can be generalised to other SP models. Generalisation of findings between different SP models has to be made with caution, as some findings are context and intervention specific and may not be transferable to other settings and interventions. Another limitation is that all eligible records were included in the review independently of their appraised methodological quality. Poor quality studies were retained because more rigorous studies are lacking. Although the quality of included studies and reports is considered to be low, they contain relevant information that could contribute to improved future practice.

## Conclusion

This review identified a range of facilitators and barriers to the implementation and delivery of SP services based in general practice involving a navigator. Some of the identified themes are similar to those of other integrated care interventions, whereas others appear to be specific to SP interventions, and SP models. Findings provide valuable and unique insights that commissioners, managers, and providers can use to guide the implementation process and delivery of SP interventions. The lack of published studies in this field and the poor methodological quality of available evidence highlight the need for rigorous and high quality studies that evaluate factors that influence the implementation and delivery of SP services.

## Additional file

Additional file 1: Demonstrating the search strategy for CINAHL (EBSCOhost). The advanced search techniques were adjusted to meet the different requirements of the included electronic databases. (DOCX 80 kb)

## Abbreviations

ASSIA: Applied Social Sciences Index& Abstracts
CCG: Clinical Commissioning Group
CINAHL: The Cumulative Index to Nursing and Allied Health Literature
GP: General Practitioner
HMIC: Health Management Information Consortium
NICE: National Institute for Health and Care Excellence
PCT: Primary Care Trust
SP: Social Prescribing
UK: United Kingdom
WEMWBS: Warwick Edinburgh Mental Wellbeing Scale

## References

[1] Marmot M, Allen J, Goldblatt P, Boyce T, McNeish D, Grady M, et al. Fair society, healthy lives. England: The Marmot Review; 2010.

[2] Brandling J, House W (2009). Social prescribing in general practice: adding meaning to medicine. Br J Gen Pract.

[3] Kimberlee, R. Developing a social prescribing approach for Bristol. Project Report. Bristol Health & Wellbeing Board; 2013.

[4] Maughan DL, Patel A, Parveen T, Braithwaite I, Cook J, Lillywhite R (2016). Primary-care-based social prescribing for mental health: an analysis of financial and environmental sustainability. Prim Health Care Res Dev.

[5] South J, Higgins TJ, Woodall J, White SM (2008). Can social prescribing provide the missing link?. Prim Heal Care Res Dev.

[6] Brandling J, House W. Investigation into the feasibility of a social prescribing service in primary care: a pilot project. Bath: University of Bath; 2007.

[7] Centre for Reviews and Dissemination (2015). Evidence to inform the commissioning of social prescribing.

[8] NHS England (2014). Five year forward view.

[9] UK Government (2016). Social value act: information and resources [internet]. Cabinet off.

[10] National Institute for Health and Care Excellence. Depression in adults: recognition and management [internet]. Clin Guidel. 2009; [cited 2016 Dec 1]. Available from: https://www.nice.org.uk/guidance/cg90 31990491

[11] Kimberlee R, Ward R, Jones M, Powell J. Proving our value: measuring the economic impact of wellspring healthy living Centre’s social prescribing wellbeing programme for low level mental health issues encountered by GP services. Bristol: University of the West of England; 2014.

[12] Kinsella S. Social prescribing. A review of the evidence. Wirral: Wirral Council Business & Public Health Intelligence Team; 2015.

[13] Thomson LJ, Camic PM, Chatterjee HJ (2015). Social prescribing: a review of community referral schemes.

[14] Kilgarriff-Foster A, O’Cathain A (2015). Exploring the components and impact of social prescribing. J Public Ment Health.

[15] Campbell M, Fitzpatrick R, Haines A, Kinmouth A, Sandercock P, Spiegelhalter D (2000). Framework for design and evaluation of complex interventions to improve health. BMJ.

[16] Moore G, Audrey S, Barker M, Bond L, Bonell C, Hardeman W, et al. Process evaluation of complex interventions. UK Medical Research Council (MRC) guidance. MRC Population Health Science Research Network; 2014.

[17] Moore G, Audrey S, Barker M, Bond L, Bonell C, Hardeman W, et al. Process evaluation of complex interventions: a summary of Medical Research Council guidance. 2014. 1–17. 10.1136/bmj.h1258 PMC4366184 25791983

[18] Peters DH, Adam T, Alonge O, Agyepong IA, Tran N. Implementation research: what it is and how to do it. BMJ. 2013;347:f6753. 10.1136/bmj.f6753 24259324

[19] RAND Europe EYL (2012). National evaluation of the Department of Health’s integrated care pilots.

[20] Ling T, Brereton L, Conklin A, Newbould J, Roland M (2012). Barriers and facilitators to integrating care: experiences from the English integrated care pilots. Int. J. Integr. Care..

[21] Durlak JA, DuPre EP (2008). Implementation matters: a review of research on the influence of implementation on program outcomes and the factors affecting implementation. Am J Community Psychol Psychol.

[22] Tong A, Flemming K, McInnes E, Oliver S, Craig J. Enhancing transparency in reporting the synthesis of qualitative research: ENTREQ. BMC Med Res Methodol. 2012;12:181. 10.1186/1471-2288-12-181 PMC3552766 23185978

[23] Rodgers M, Sowden A, Petticrew M, Arai L, Roberts H, Britten N (2009). Testing methodological guidance on the conduct of narrative synthesis in systematic reviews. Evaluation.

[24] Popay J, Robers H, Sowden A, Petticrew M, Arai L, Rodgers M (2006). Guidance on the conduct of narrative synthesis in systematic reviews.

[25] Pluye P, Robert E, Cargo M, Bartlett G, O’Cathain A, Griffiths A (2011). Proposal: a mixed methods appraisal tool for systematic mixed studies reviews [internet].

[26] Pope C, Mays N, Popay J (2007). Synthesising qualitative and quantitative health evidence: a guide to methods.

[27] Moher D, Liberati A, Tetzlaff J, Altman D (2009). Preferred reporting items for systematic reviews and meta-analyses: the PRISMA statement. J Clin Epidemiol United States.

[28] Polley M, Dixon M, Pilkington K, Ridge D, Herbert N, Fleming J (2016). Report of the annual social prescribing network conference.

[29] Brandling J, House W, Howitt D, Sansom A (2011). “New routes”: pilot research project of a new social prescribing service provided in Keynsham.

[30] ERS Research and Consultancy (2013). Newcastle social prescribing project final report august 2013.

[31] Farenden C, Mitchell C, Feast S, Verdenicci S (2015). Community navigation in Brighton & Hove. Evaluation of a social prescribing pilot.

[32] Friedli L, Themessl-huber M, Butchart M (2012). Evaluation of Dundee equally well sources of support: social prescribing in Maryfield.

[33] The Health Foundation (2015). Shine 2014 final report social prescribing: integrating GP and community assets for health.

[34] Age UK. Social prescribing. A model for partnership working between primary care and the voluntary sector. http://www.ageconcernyorkshireandhumber.org.uk/uploads/files/Social%20Prescribing%20Report%20new.pdf.

[35] Dayson C, Bashir N, Pearson S (2013). From dependence to independence: emerging lessons from the Rotherham social prescribing pilot.

[36] Hjelmar U, Hendriksen C, Hansen K. Motivation to take part in integrated care - an assessment of follow-up home visits to elderly persons. Int J Integr Care. 2011;11:e122. 10.5334/ijic.649 PMC3564424 23390410

[37] MacAdam M (2008). Frameworks of integrated care for the Eederly: a systematic review.

[38] Chaudoir SR, Dugan AG, Barr CH. Measuring factors affecting implementation of health innovations: a systematic review of structural, organizational, provider, patient, and innovation level measures. Implement Sci. 2013;8:22. 10.1186/1748-5908-8-22 PMC3598720 23414420

[39] Mc Hugh S, O’Mullane M, Perry IJ, et al. Barriers to, and facilitators in, introducing integrated diabetes care in Ireland: a qualitative study of views in general practice. BMJ Open 2013;3:e003217. 10.1136/bmjopen-2013-003217 PMC3753479 23959754

