# Supplementary material for: Facilitators and barriers of implementing and delivering social prescribing services: a systematic review
Source: BMC Health Serv Res. 2018 Feb 7;18:86. doi: 10.1186/s12913-018-2893-4 (PMC5803993; doi:10.1186/s12913-018-2893-4)
Supplement: Additional file 1: — Demonstrating the search strategy for CINAHL (EBSCOhost). The advanced search techniques were adjusted to meet the different requirements of the included electronic databases. (DOCX 80 kb) [file 12913_2018_2893_MOESM1_ESM.docx]

Additional file 1

The advanced search techniques were adjusted to meet the different requirements of the included electronic databases.

Box 1. Demonstrating the search strategy for CINAHL (EBSCOhost)

| **Search 1**  social N1 prescri* OR community N1 prescri* OR “community referral” OR “community referrals” OR “social referral” OR “social referrals” OR “social intervention” OR “social interventions” OR “linking scheme” OR “linking schemes” OR exercise N2 prescription OR books N2 prescription OR arts N2 prescription OR prescription N2 learning OR education N2 prescription  **Search 2**  “primary care” OR “primary-care” OR “health care” OR healthcare OR “health-care” OR “general practice” OR “PCT” OR “National Health Service” OR “NHS” OR “health centres” OR “health centre” OR “health services” OR “health service” OR “social care”  **Search 3**  UK OR “United Kingdom” OR England OR “Northern Ireland” OR Scotland OR Wales OR “Great Britain”  **Search 4**  Health OR wellbeing OR “well-being”  **Search 5**  Search 1 AND Search 2 AND Search 3 AND Search 4 |
| --- |

The NX operator indicates within X words

* Indicates truncation to find all forms of that word

“” Indicates that the search engine looks for words in the exact order
